# Tetramethylpyrazine enhances neuroprotection and plasticity in cerebral ischemia-reperfusion injury via RhoA/ROCK2 pathway inhibition

**DOI:** 10.3389/fphar.2025.1594283

**Published:** 2025-05-27

**Authors:** Yixin Zhang, Xin Zhang, Xiaocheng Shi, Weijing Liao, Junbin Lin

**Affiliations:** Department of Rehabilitation Medicine, Zhongnan Hospital of Wuhan University, Wuhan, China

**Keywords:** tetramethylpyrazine, cerebral ischemia-reperfusion injury, neuroplasticity, neuroprotection, nature products

## Abstract

Tetramethylpyrazine (TMP) is an active component of the Chuanxiong, effectively crosses blood-brain barrier (BBB). It exhibits neuroprotective potential in cerebral ischemia-reperfusion injury (CIRI). This study performed middle cerebral artery occlusion/reperfusion (MCAO/R) surgery in rats to evaluate TMP’s efficacy and mechanisms in mitigating CIRI. Rats received intraperitoneal TMP (40 mg/kg) for 3 days prior to MCAO/R and continued for 14 days post-surgery. Behavioral tests were conducted using mNSS and Morris water maze tests. Histopathological analyses, including HE, Nissl, and TUNEL staining. mRNA sequencing revealed that RhoA and ROCK2 were upregulated in the CIRI model and downregulated by TMP treatment. GO enrichment and KEGG enrichment showed RhoA and ROCK were related to neuroplasticity. Western blot and immunofluorescence staining confirmed that TMP inhibited RhoA, ROCK2, phosphorylated LIMK, and phosphorylated cofilin expression. Additionally, TMP increased the levels of neuroplasticity-related proteins PSD95 and MAP2, promoting synaptic and dendritic regeneration. Administration of lysophosphatidic acid (LPA), a RhoA/ROCK pathway agonist, attenuated TMP’s neuroprotective effects, validating the pathway’s role in TMP-mediated protection. These findings indicate that TMP confers neuroprotection in CIRI by inhibiting the RhoA/ROCK pathway and enhancing neuroplasticity, underscoring its therapeutic potential in CIRI.

## 1 Introduction

As the world’s second deadliest health condition, stroke-related healthcare costs - including acute treatment and prolonged recovery processes - create considerable financial pressures on both individuals and healthcare systems (GBD, 2019 Stroke Collaborators, 2021). Approximately 87% of stroke is ischemic stroke and 13% is hemorrhagic stroke (Virani et al., 2020). Current therapeutic options for the acute phase of ischemic stroke include pharmacological thrombolysis and mechanical thrombectomy. However, due to time window limitations, many patients are unable to receive timely treatment, and only 2%–10% of patients worldwide are eligible for these therapies (Mokin et al., 2019; Hacke et al., 2008). Thus, the development of novel therapeutic strategies for ischemic stroke remains imperative.

Although timely reperfusion is essential for the restoration of neurological function, cerebral ischemia-reperfusion injury (CIRI) impedes the recovery process following ischemic stroke. CIRI leads to cellular excitotoxicity, Ca^2+^ overload, autophagy, and aberrant immune responses and so on (Zhou et al., 2018; Campbell et al., 2019). At the same time, CIRI also causes morphological changes in neurons, such as dendritic and axonal retraction and loss of synapses and dendritic spines (Brown and Murphy, 2008). A series of damages to brain can lead to patients experiencing impaired or even loss of normal function. Therefore, how to treat CIRI has been a hot spot in research. Currently, the drugs used to treat CIRI mainly include anti-free radical damage drugs (Takase et al., 2018), anti-inflammatory drugs (Xu et al., 2018), Ca^2+^ antagonists (Carlson et al., 2020), excitatory amino acid receptor antagonists (Margaill et al., 1996), and traditional Chinese medicine (TCM) (Fan et al., 2020).

TCM, renowned for its multi-component and multi-target approaches with minimal side effects, has been utilized for centuries in China and other countries to treat ischemic stroke, with Chuanxiong—a widely used and ancient medicinal herb from Sichuan—first documented in the “Shen Nong’s Canon of Materia Medica (Chen et al., 2018). Chuanxiong has been used to protect against ischemic injury, enhance immunity, and treat menstrual cramps and diabetes (Wang J. et al., 2022). In clinical treatment, Chuanxiong has also been made into a variety of Chinese medicines, including ligustrazine injection, Xuefu Zhuyu Granules and Chuanxiong Chatiao San, etc (Shao et al., 2021; Li et al., 2021; Wang et al., 2019). Tetramethylpyrazine (TMP), one of the components of Chuanxiong, effectively penetrates blood-brain barrier (BBB) (Tsai and Liang, 2001), thereby being widely utilized in central nervous system (CNS) diseases. Tetramethylpyrazine (TMP) exerts neuroprotective effects by inhibiting calcium-ion overload (Yu et al., 2019), inhibition of inflammatory response (Shah et al., 2019), promotion of BBB repair (Gong et al., 2019), enhancement of synaptic plasticity (Lin et al., 2021), and inducing recovery of neurovascular units (Feng et al., 2023). Cellular experiments have demonstrated that TMP reduces neuronal death induced by oxygen-glucose deprivation (OGD) (Shao et al., 2017), and its efficacy has been validated in clinical trials (Ni et al., 2013; Zhang et al., 2020; Yang et al., 2012).

Ischemic-hypoxic injury in the CNS activates the RhoA/ROCK signaling pathway, where ROCK, as the primary downstream effector of RhoA, is critical in cytoskeletal regulation (Lang et al., 1996; Matsui et al., 1996; Nakagawa et al., 1996; Wang Q. et al., 2022). ROCK participates in multiple processes such as cytoskeletal regulation and cell survival by phosphorylating LIM kinase (LIMK) (Mulherkar and Tolias, 2020), which subsequently phosphorylates cofilin, thereby modulating F-actin homeostasis and inducing cytoskeletal remodeling that leads to dendritic contraction and the loss of dendritic spines (Jahani et al., 2018; Allen et al., 2010). TMP can inhibit the RhoA/ROCK signaling pathway and alleviate damage to brain microvascular endothelial cells (Yang et al., 2017). Leveraging mRNA sequencing analysis, this study further explores the neuroprotective effects of TMP in CIRI, thereby providing theoretical foundation for the application of TMP.

## 2 Materials and methods

### 2.1 Experimental animal preparation

**FIGURE 1:**
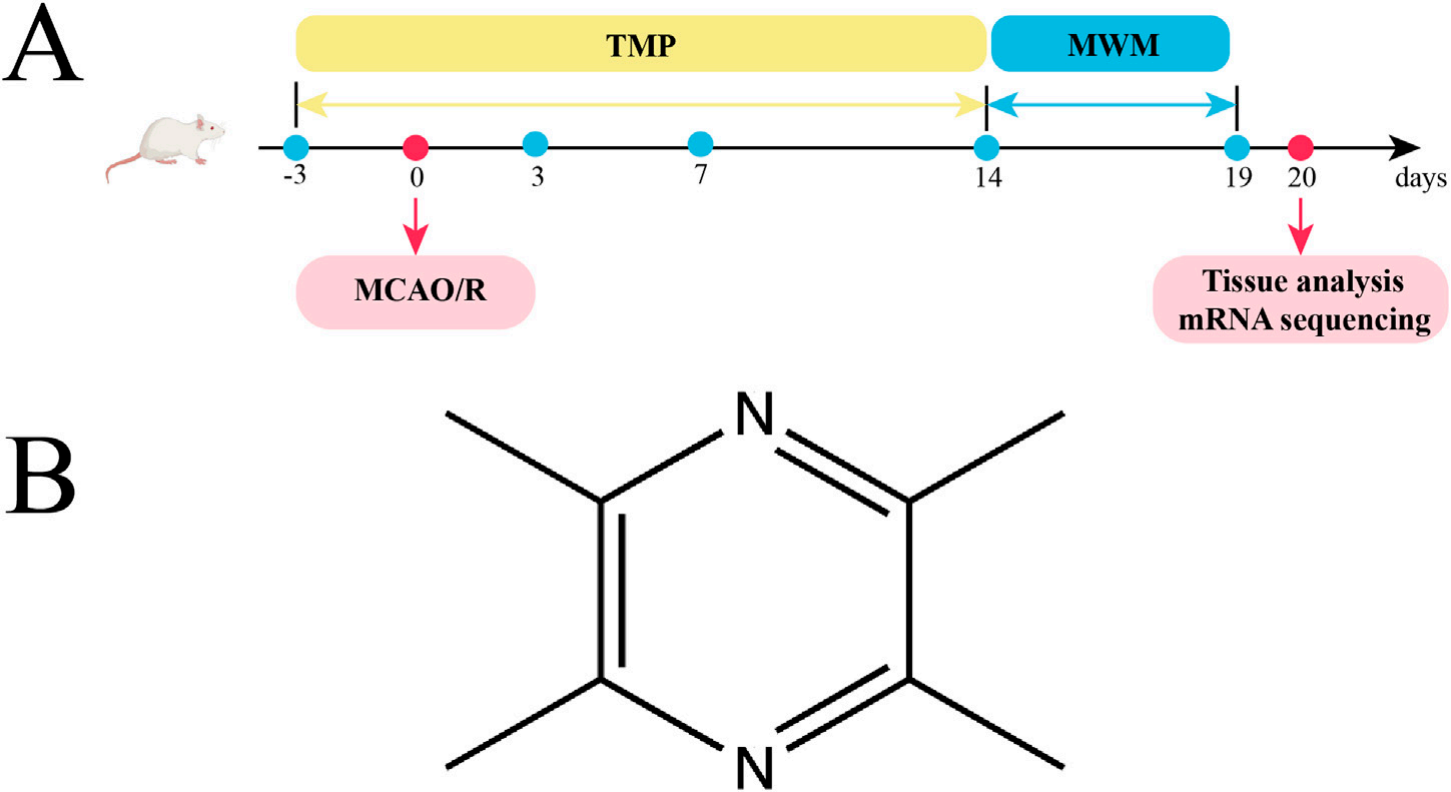
Experimental protocol, and TMP. **(A)** TMP was injected intraperitoneally 3 days before MCAO/R surgery and 14 days after it. Behavioral test was conducted at postoperative days (POD) 3, 7, and 14. The Morris water maze (MWM) was tested from POD14 to POD19. Tissue analysis and mRNA sequencing were performed on day 20. **(B)** Chemical structural formula of TMP.

We utilized Sprague-Dawley rats (n = 64) sourced from an AAALAC-accredited supplier (Hubei Bainter Laboratory Animal Technology Co., China). Ethical oversight was maintained through formal review (Approval ID: WP20230166) by Zhongnan Hospital, Wuhan University. After acclimatizing the rats for 3 days, they were divided into the sham, model, and TMP groups. To allow the drug concentration to build up, the rats in TMP group were injected with TMP (40 mg/kg) for 3 days before the surgery. The specific experimental steps were: after anesthetizing the rats with 3% pentobarbital sodium, the skin was prepared and sterilized. A 1.5–2 cm notch was cut in the neck of the rats. After separating the muscle, the common carotid artery (CCA) was exposed. The proximal CCA and external carotid artery (ECA) were ligated separately. A thin thread was left in the CCA to facilitate subsequent fixation of filament. Transient focal ischemia was induced via intraluminal filament technique. After temporary occlusion of the CCA with a 40 g vascular clamp, a 4–0 nylon suture (diameter 0.23 ± 0.02 mm) coated with poly-L-lysine was introduced through a micro-incision (0.4 mm) and navigated 20.5 ± 0.3 mm into the internal carotid artery to achieve MCAO. For sham group, no filament was inserted. 90 min later, the filament was removed. The Longa scale was performed by an uninformed researcher (Longa et al., 1989). Subjects meeting predefined inclusion criteria (neurological deficit scores 1–3 on the 5-point Garcia scale) were prospectively enrolled for longitudinal assessment, while those scoring at scale extremes (0 = no deficit; 4 = maximal impairment) were systematically eliminated to ensure cohort homogeneity. The experimental steps are shown in Figure 1.

### 2.2 Behavioral tests

Behavioral changes in different groups were assessed using the mNSS and the MWM test (n = 10). The mNSS was performed on postoperative days 3, 7, and 14. The mNSS, totaling 18 points, was assessed from sensory, motor, balance, and reflexes (Bieber et al., 2019).

The MWM was performed on postoperative days 14–19. The water was dyed black with ink to track the rat’s movement. An animal video tracking was used to record the rats’ swimming trajectories, speeds, arrival times at the platform, and the number of times they traversed the platform. MWM test was divided into a 5-day spatial learning period and a platform-free exploration period on the last day. During the spatial learning period, the platform was placed in one quadrant. The rats were placed in the water from four positions. If the rats did not find the platform within 60 s, the rats were instructed to stand on the platform to ensure that all rats had the same time for spatial observation and learning. On the sixth day, the platform was removed and the rats were placed in the water for 60 s to end the experiment.

### 2.3 HE and nissl staining

Following induction of deep anesthesia using sodium pentobarbital (n = 5), cardiac perfusion was performed transcardially with 4% paraformaldehyde (PFA). Excised cerebral tissues underwent 24-hour post-fixation in the same fixative at 4°C before being processed for paraffin embedding. Using a rotary microtome, consecutive 5 μm coronal sections were obtained and mounted on adhesive-coated slides. After standard dewaxing and rehydration procedures, adjacent sections were alternately processed for HE and Nissl staining to assess cytoarchitectural integrity. A systematic random sampling approach was implemented for microscopic analysis, with four non-overlapping fields (400 × magnification) examined in peri-lesional regions per animal across three anatomically matched sections. Brightfield imaging was conducted utilizing a BX53 light microscope (Olympus) equipped with a DP74 digital camera system. To maintain objectivity, experimental groups were coded prior to analysis, and quantitative assessments were performed by two independent investigators unaware of treatment conditions.

### 2.4 TUNEL staining

Following standard paraffin section preparation as previously described, apoptotic cells were identified using TUNEL assay system. Nuclear visualization was achieved through DAPI fluorescent labeling. For quantitative analysis, a systematic random sampling strategy was employed, wherein four non-overlapping microscopic fields (400 × magnification) within peri-infarct regions were captured per animal using a BX53 fluorescence microscope (Olympus) equipped with appropriate filter sets for DAPI (excitation/emission: 358/461 nm) and TUNEL detection. Three anatomically matched coronal sections were analyzed for each experimental subject. To ensure unbiased data collection, all image acquisition and analysis procedures were conducted by investigators blinded to experimental conditions, with sample identification codes maintained by an independent researcher until completion of quantitative assessments.

### 2.5 mRNA sequencing

To detect the impact of TMP on the cerebral gene expression, we took the tissues of the ischemic penumbra from rats in the sham, model and TMP groups (n = 3) respectively. After RNA extraction using the Trizol method, the concentration and integrity of the RNA were assessed. Library preparation was performed using the Library Prep Kit (BGI-Shenzhen, China). The library was amplified to obtain DNA nanoballs (DNBs). The sequencing data underwent filtering and quality control with SOAPnuke (v2.2.1) (Li et al., 2008), ensuring only high-quality reads were retained for further analysis. For alignment against the reference genome, we employed HISAT2 to accurately map the cleaned reads (Kim et al., 2015). To assess gene expression, clean reads were aligned to a database of reference coding genes utilizing Bowtie2 (Langmead and Salzberg, 2012). Gene expression quantification was then conducted using RSEM (Li and Dewey, 2011). Ultimately, to identify differentially expressed genes (DEGs), an analysis was carried out with DESeq2 (v1.34.0), applying a threshold of a q-value below 0.05 for stringent statistical significance (Love et al., 2014). To gain deeper insights into the functions and pathways of DEGs regulated by TMP in CIRI, we employed Gene Ontology (GO) enrichment and Kyoto Encyclopedia of Genes and Genomes (KEGG) pathway analysis.

### 2.6 Western blot

Following deep anesthesia induction with sodium pentobarbital (n = 3 Sprague-Dawley rats), cerebral tissues from peri-infarct regions were harvested after transcardial perfusion with ice-cold phosphate-buffered saline. Tissue specimens (50 mg) were processed through mechanical homogenization using RIPA extraction buffer. Protein quantification was subsequently performed via BCA protein assay kit prior to SDS-PAGE separation. Based on the predicted molecular weights of target proteins, protein extracts were resolved on discontinuous SDS-polyacrylamide gels (Epizyme Biomedical, Shanghai, China) under reducing conditions. Electrophoretically separated proteins were transferred to PVDF membranes (Millipore). Bands were blocked for 1 h. Subsequently, the strips were incubated with primary antibodies: anti-RhoA protein (RhoA; 1:1,000; Proteintech), anti-ROCK2 protein (ROCK2; 1:1,000; Abmart), anti-phosphorylated LIMK1 protein (Phospho-LIMK1; 1:1,000; Abmart), anti-phosphorylated Cofilin protein (Phospho-Cofilin; 1:1,000; Abmart), anti-PSD95 protein (PSD95; 1:2000; Proteintech), anti-MAP2 protein (MAP2; 1:2000; Affinity), and anti-β actin protein (β actin; 1:4,000. Proteintech). Then the strips were placed in secondary antibody and incubated for 1 h. Protein bands were imaged on Bio-Rad. All experimental groups were coded prior to analysis, and quantitative assessments were conducted by investigators blinded to treatment conditions to ensure unbiased data interpretation.

### 2.7 Immunofluorescence staining

Paraffin sections were prepared as above. After antigen repair with EDTA antigen repair solution, the sections were blocked with blocking buffer (n = 5). Specific primary antibodies were added overnight. After washing the sections, specific secondary antibody was added in the dark. In the end, sections were stained with DAPI. The primary antibodies were: anti-RhoA protein (RhoA; 1:800; Proteintech), anti-ROCK2 protein (ROCK2; 1:200; Abmart), anti-phosphorylated LIMK protein (Phospho-LIMK1; 1:500; Abmart), anti-phosphorylated Cofilin protein (Phospho-Cofilin; 1:500; Abmart), anti-PSD95 protein (PSD95; 1:800; Proteintech), and anti-MAP2 protein (MAP2; 1:200; Affinity).For quantitative histological analysis, a systematic random sampling approach was implemented in peri-infarct cortical regions. Four non-overlapping microscopic fields (400 × magnification) were captured per animal using an Olympus BX53 imaging system equipped with a DP74 digital camera (Olympus Corporation, Tokyo, Japan). Three anatomically matched coronal sections, spaced at 200 μm intervals to ensure independent sampling, were analyzed for each experimental subject. To maintain objectivity throughout the quantification process, all image acquisition and analysis procedures were performed by investigators blinded to experimental conditions, with sample identification codes maintained by an independent researcher until completion of data collection.

### 2.8 Observation of synaptic structure

Following deep anesthesia induction via intraperitoneal administration of sodium pentobarbital (n = 5), cubic tissue blocks (1 mm^3^) from peri-infarct cortical regions were dissected and immersion-fixed in 2.5% glutaraldehyde/4% paraformaldehyde. Following fixation with 1% osmium tetroxide, tissue specimens underwent sequential ethanol dehydration and subsequent embedding in Epon 812 epoxy resin. Ultrathin sections were obtained using a diamond knife-equipped ultramicrotome, followed by dual staining with uranyl acetate and lead citrate for electron density enhancement. Ultrastructural examination was performed using a Hitachi HT7800 TEM. A systematic random sampling protocol was implemented, wherein four non-overlapping fields (15,000 × magnification) within the ischemic penumbra were captured per animal across three anatomically matched tissue planes spaced at 200 µm intervals. To ensure unbiased evaluation, all specimens were coded prior to imaging, and quantitative morphometric analyses were performed by researchers blinded to groupings using ImageJ software with particle analysis plugins.

### 2.9 Golgi staining

After rats (n = 5) were anesthetized, brain tissues were removed as quickly as possible, handling carefully so as not to damage or compress the tissues. The blood on the surface of the tissues was quickly rinsed off with double-distilled water (ddH_2_O), and the tissues were subsequently cut into 100 μm thin slices. The sections were dehydrated and sealed with a resin sealer after completing the subsequent staining. Dendritic length and dendritic spine density were analyzed separately using Sholl analysis in ImageJ software.

### 2.10 Data analysis

The data were analyzed using GraphPad Prism 8.0. mNSS and the escape latency data were tested using two-way ANOVA, and the rest of the data were tested using one-way ANOVA. Statistical significance was established at P values below the 0.05 threshold.

## 3 Results

### 3.1 TMP improves neurological function in rats after CIRI

**FIGURE 2:**
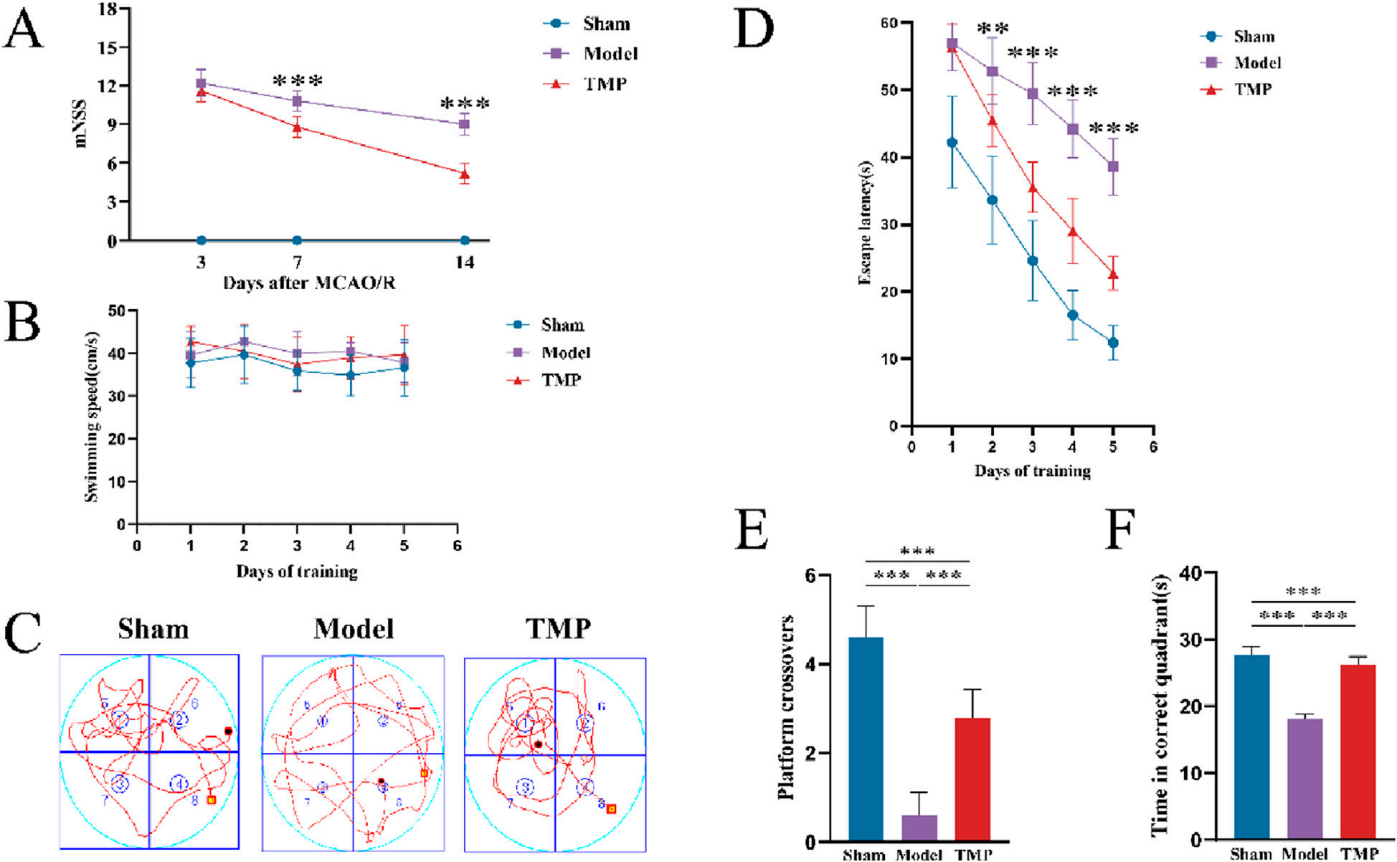
TMP attenuated neurological deficits after CIRI. **(A)** Comparison of mNSS between Model and TMP groups. **(B)** Swimming speed of rats. **(C)** Swimming trails of rats. **(D)** Escape latency time. **(E)** Number of times crossed the original platform. **(F)** Time in correct quadrant *n* = 10. Data are expressed as mean ± SD. **P < 0.01, ***P < 0.001.

To evaluate the impact of TMP on behavioral changes, we implemented the mNSS and MWM tests. After performing the mNSS on days 3, 7, and 14, we found that the rats in the sham group had mNSS of 0, indicating no neurological deficits. On days 7 and 14, the mNSS scores of rats in the TMP group were significantly lower than those in the model group (Figure 2A; *P* < 0.01). It suggests although TMP could not completely eliminate the neurological impairments, it was effective in improving the neurological functions over time.

From days 14 to day 19, we conducted MWM tests. The results showed no significant difference in swimming speed between different groups, which eliminated any potential bias caused by differences in motor ability (Figure 2B). The escape latency time gradually decreased in all groups as training progressed. Starting from the second day, the TMP group showed a shorter escape latency time than model group (Figure 2D; *P* < 0.01), and this difference became more significant as time went on (Figure 2D; *P* < 0.001). After completing the 5-day learning period, we conducted the spatial exploration test to measure the time rats spent in the correct quadrant, the number of times they crossed the platform. The TMP group crossed the platform significantly more times than the model group (Figure 2E; *P* < 0.001), and they also spent significantly more time in the correct quadrant than model group (Figure 2F; *P* < 0.001).

### 3.2 TMP ameliorates neuronal damage in rats after CIRI

**FIGURE 3:**
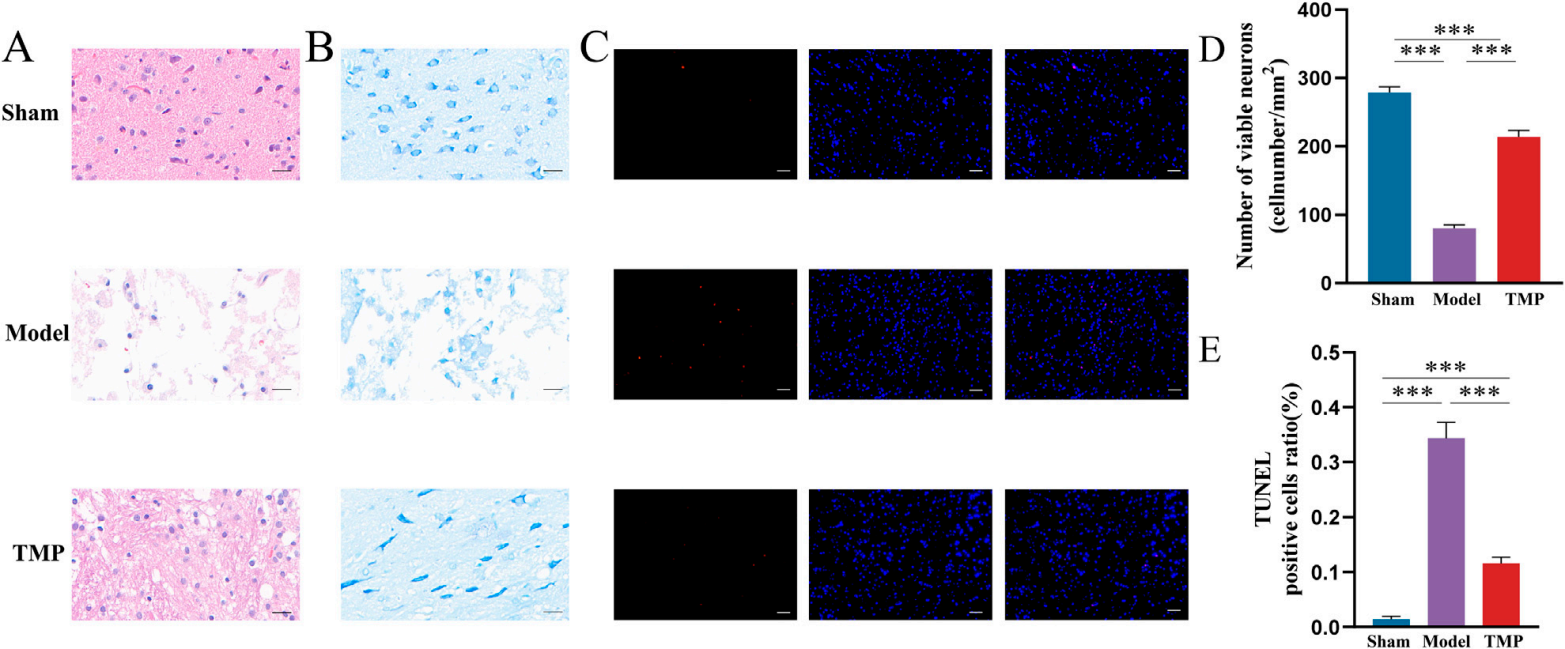
TMP exerts neuroprotective effects after CIRI injury. **(A)** Images of HE staining. **(B,D)** Results of Nissl staining. **(C,E)** Results of TUNEL staining. *n* = 5. ***P < 0.001.

To evaluate the impact of TMP on neurons in peri-infarct area, we performed HE, Nissl and TUNEL staining to assess neuronal damage. The results of HE staining showed the peri-infarct tissues in the sham group were structurally intact, uniformly arranged and clearly outlined, with bluish-purple nuclei and light red cytoplasm. In the model group, the cells exhibited obvious defects and disordered arrangement. Some cells were swollen, and the nuclei were solidified in the model group. Although necrotic cells were still present in the TMP group, the overall number of surviving cells were more, and edema was reduced (Figure 3A).

The results of Nissl staining showed the neurons in sham group were well-arranged and stained deeply, with clear nuclei and nucleoli and visible Nissl bodies. Whereas the model and TMP groups had shallow staining, nuclear consolidation or fragmentation, and some Nissl bodies disappeared (Figure 3B). Further counting of surviving neurons revealed that there were significantly more surviving neurons in the TMP group than in the model group (Figure 3D; *P* < 0.001).

TUNEL staining specifically shows broken DNA, therefore it is used to detect cell death. Red color after staining indicates positive cells, and blue color indicates nuclei. There are no positive cells were observed in the sham group, and positive cells were seen in both the model and TMP groups (Figure 3C). Statistical analysis showed that the number of positive cells in the TMP group was significantly less than that in the model group (Figure 3E; *P* < 0.001). These results suggested that TMP promoted the repair of neurons.

### 3.3 mRNA-sequencing results suggest RhoA/ROCK2 as a potential target of TMP to protect against CIRI

**FIGURE 4:**
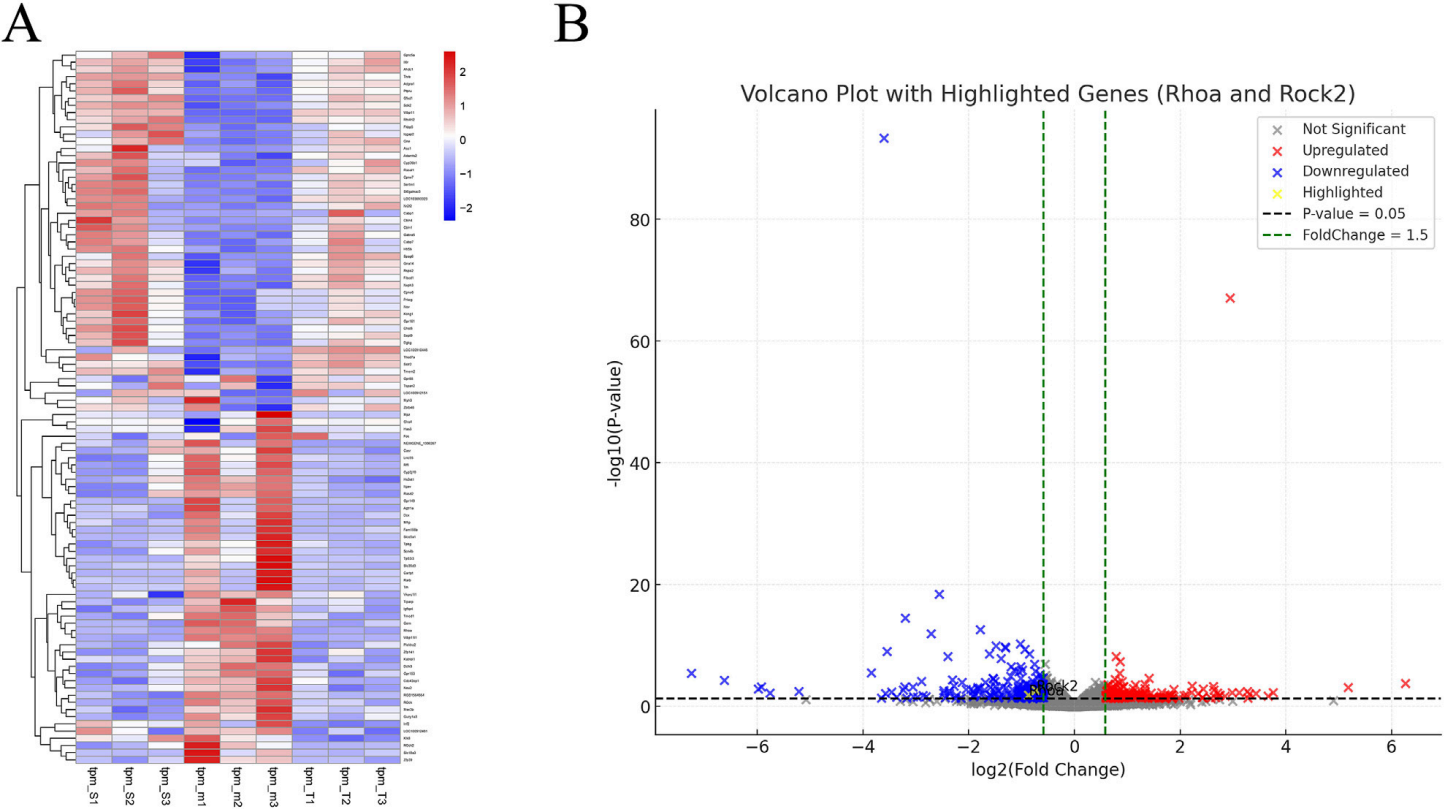
Differentially Expressed Genes **(A)** Heat map of DEGs. **(B)** Volcano plot of DEGs.

After TMP intervention, we extracted RNA from the peri-infarct area from sham, model and TMP groups for mRNA sequencing. We filtered the top 100 DEGs by *P*-value from smallest to largest (Figure 4A) and found that RhoA and ROCK2 were up-regulated in the model group compared to the sham group, but TMP intervention resulted in significant down-regulation of RhoA and ROCK. This indicates that RhoA and ROCK2 may be the pathogenic genes of CIRI and the target genes of TMP intervention. Additionally, we found 391 DEGs between model and TMP groups, including 172 upregulated genes and 219 downregulated genes (Figure 4B).

### 3.4 GO analysis

**FIGURE 5:**
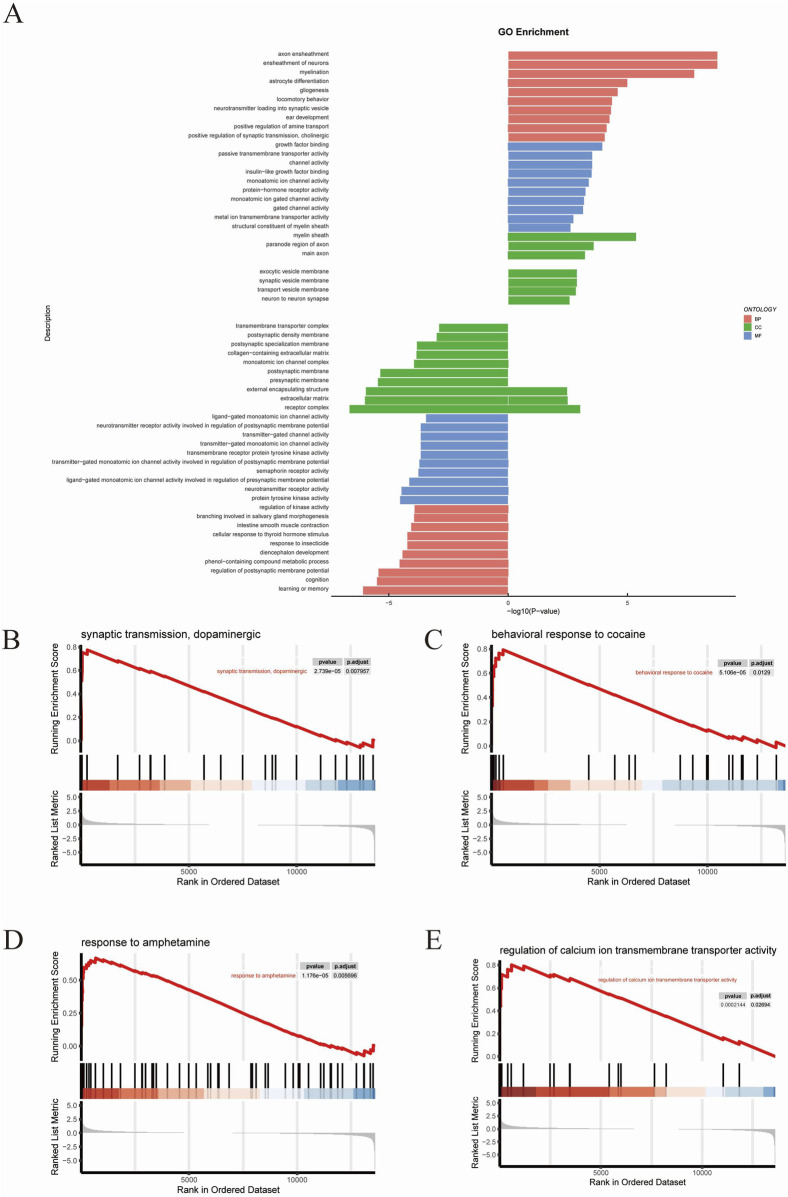
GO analysis **(A)** The results of GO analysis. **(B–E)** The results of GSEA analysis.

GO analysis was performed on 391 DEGs to predict the potential biological functions of DEGs between TMP and model groups. GO functional enrichment revealed that the top 10 BPs upregulated in the TMP group were axon ensheathment, ensheathment of neurons, myelination, astrocyte differentiation, gliogenesis, locomotory behavior, neurotransmitter loading into synaptic vesicles, ear development, positive regulation of amine transport, positive regulation of synaptic transmission, along with cholinergic processes. The top 10 enriched CCs upregulated in the TMP group were myelin sheath, paranode region of axon, main axon, exocytic vesicle membrane, synaptic vesicle membrane, transport vesicle membrane, neuron to neuron synapse, external encapsulating structure, extracellular matrix and receptor complex. The top 10 enriched MFs upregulated in TMP group were growth factor binding, passive transmembrane transporter activity, channel activity, insulin−like growth factor binding, monoatomic ion channel activity, protein−hormone receptor activity, monoatomic ion gated channel activity, gated channel activity, metal ion transmembrane transporter activity, and structural constituent of myelin sheath (Figure 5A). Based on the results of the GO functional enrichment analysis, it is evident that most of the results are related to neuroplasticity. Subsequently gene set enrichment analyses (GSEAs) were conducted (Figures 5B–E). The results revealed that the RhoA/ROCK2 signaling pathway was significantly related to dopaminergic synaptic transmission, behavioral responses to cocaine and amphetamine, and regulation of calcium ion transmembrane transport activity.

### 3.5 KEGG analysis of DEGs

**FIGURE 6:**
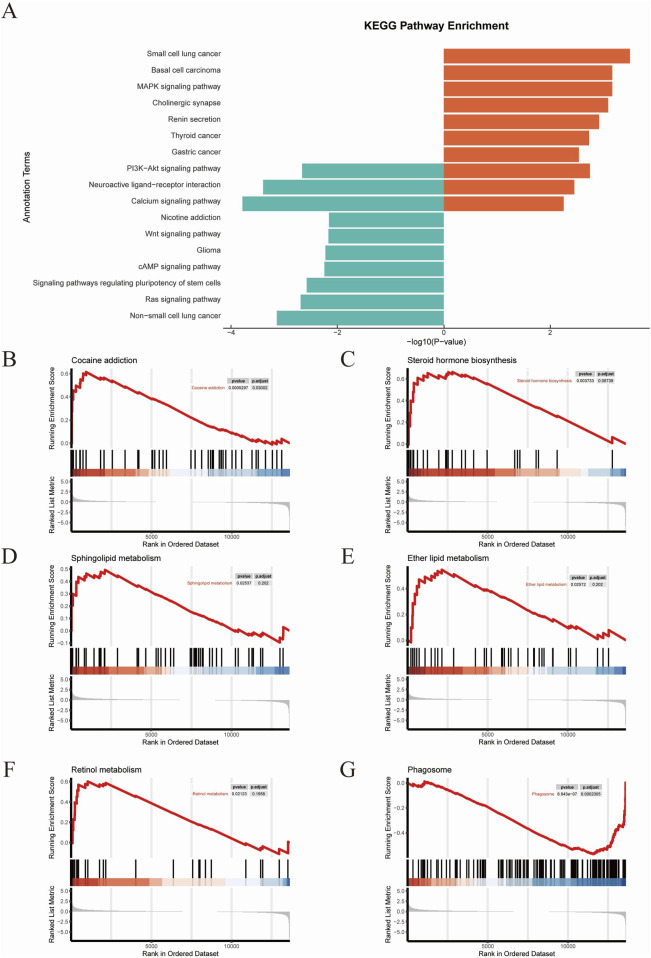
KEGG analysis **(A)** The results of KEGG analysis. **(B–G)** GSEA results.

The top KEGG pathways included the PI3K−Akt signaling pathway, Neuroactive ligand−receptor interaction, Calcium signaling pathway, Nicotine addiction, Wnt signaling pathway, Glioma, cAMP signaling pathway, signaling pathways regulating pluripotency of stem cells, Ras signaling pathway, and Non−small cell lung cancer (Figure 6A). Subsequently we performed GSEA analysis (Figures 6B–G). The results revealed RhoA/ROCK signaling pathway exhibited significant changes in several pathways that are closely related to neuroplasticity, such as Cocaine addiction, Sphingolipid metabolism, Ether lipid metabolism, Steroid hormone biosynthesis, Retinol metabolism and Phagosome.

### 3.6 TMP inhibits the expression of RhoA/ROCK signaling pathway in rats after CIRI

**FIGURE 7:**
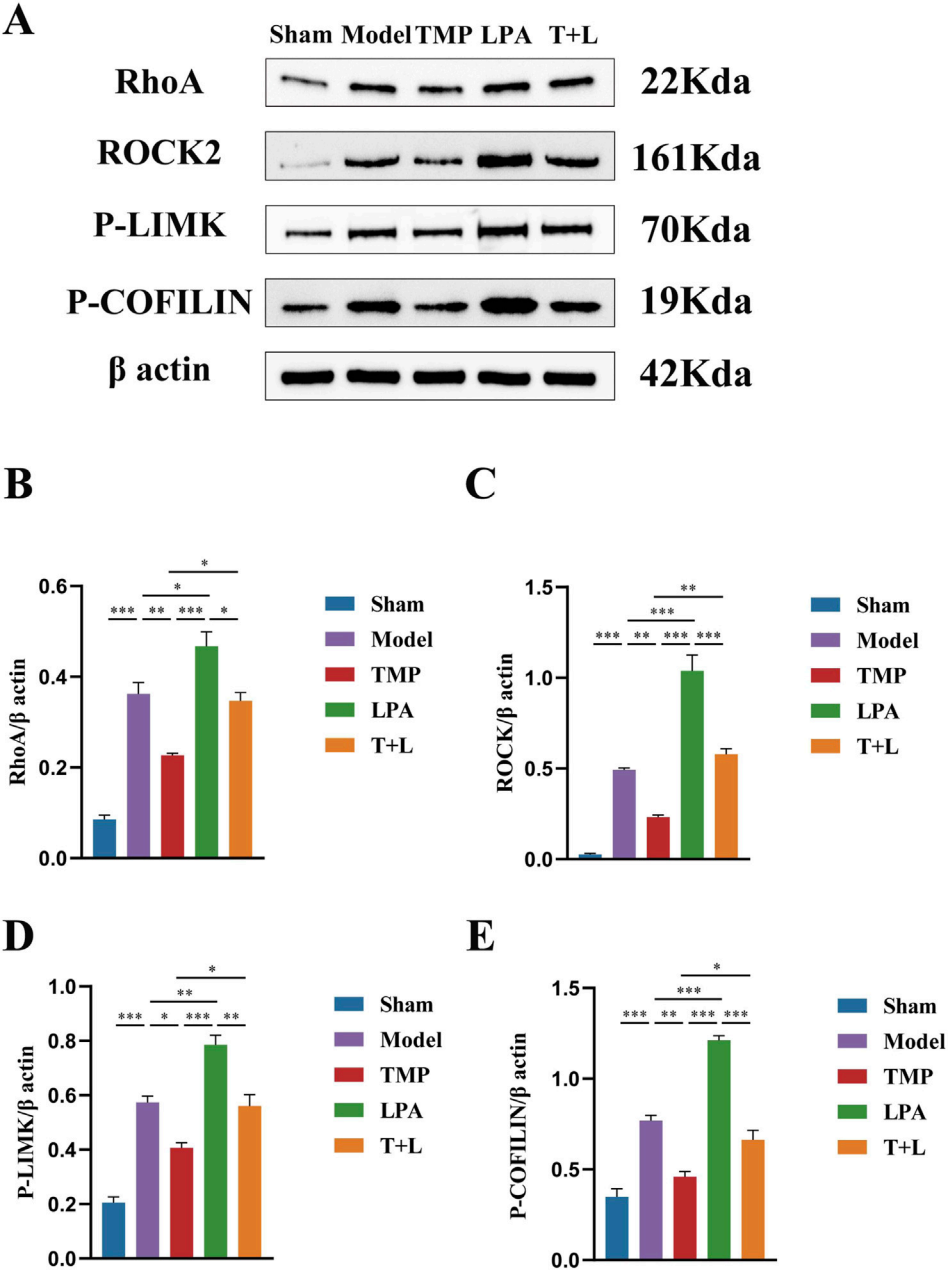
Western blot results of RhoA/ROCK signaling pathway in different groups. **(A)** Images for Western blot of RhoA, ROCK2, P-LIMK, P-COFILIN and β actin expressions. **(B)** RhoA protein expression analysis. **(C)** ROCK2 protein expression analysis. **(D)** P-LIMK protein expression analysis. **(E)** P-COFILIN protein expression analysis *n* = 3. *P < 0.05, **P < 0.01, ***P < 0.001.

**FIGURE 8:**
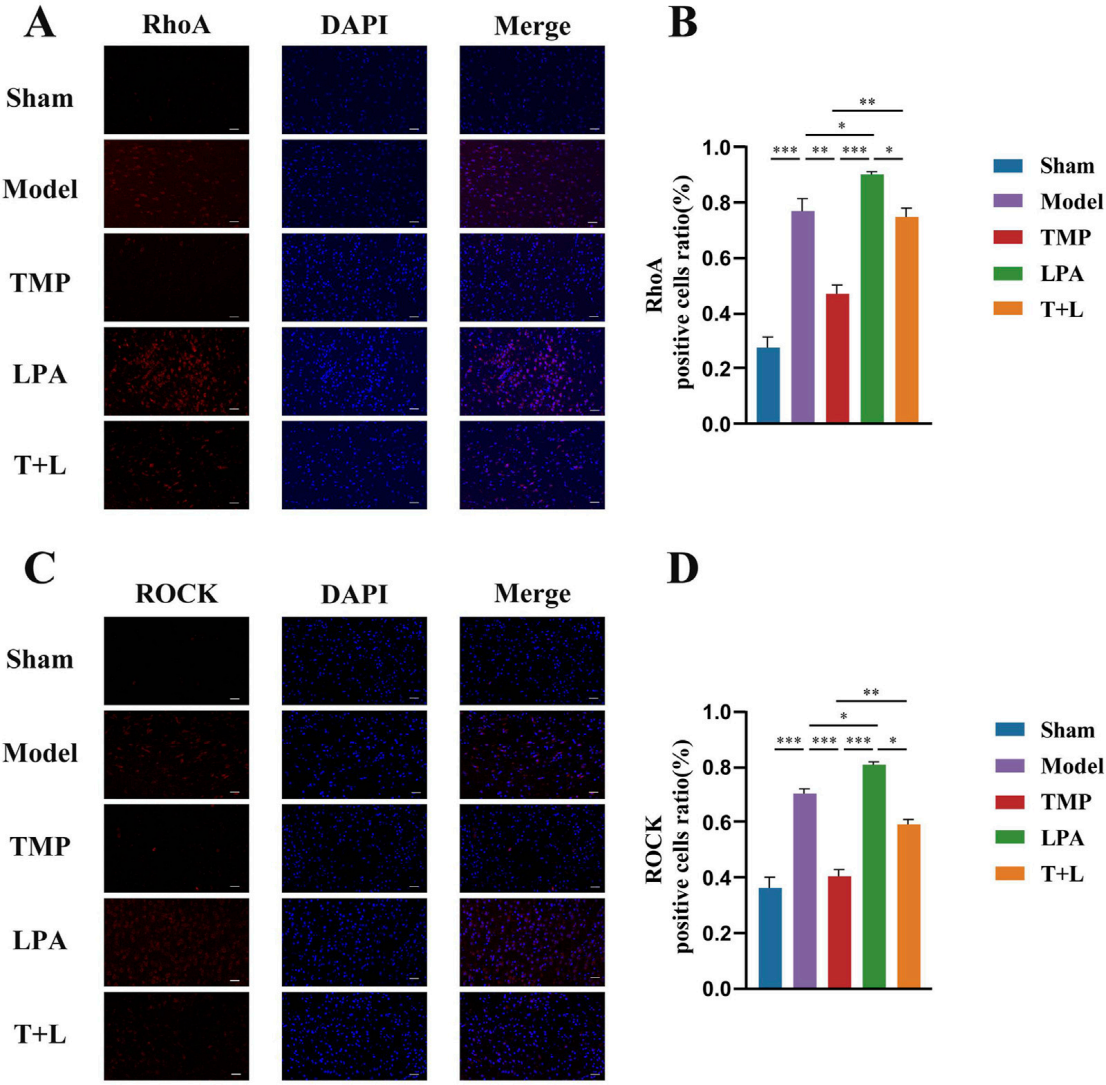
Immunofluorescence staining results of RhoA and ROCK2 in different groups. **(A)** Immunofluorescence images of RhoA. **(B)** Analysis of RhoA positive cells. **(C)** Immunofluorescence images of ROCK2. **(D)** Analysis of ROCK2 positive cells *n* = 5. Scale bar = 50 μm *P < 0.05, **P < 0.01, ***P < 0.001.

**FIGURE 9:**
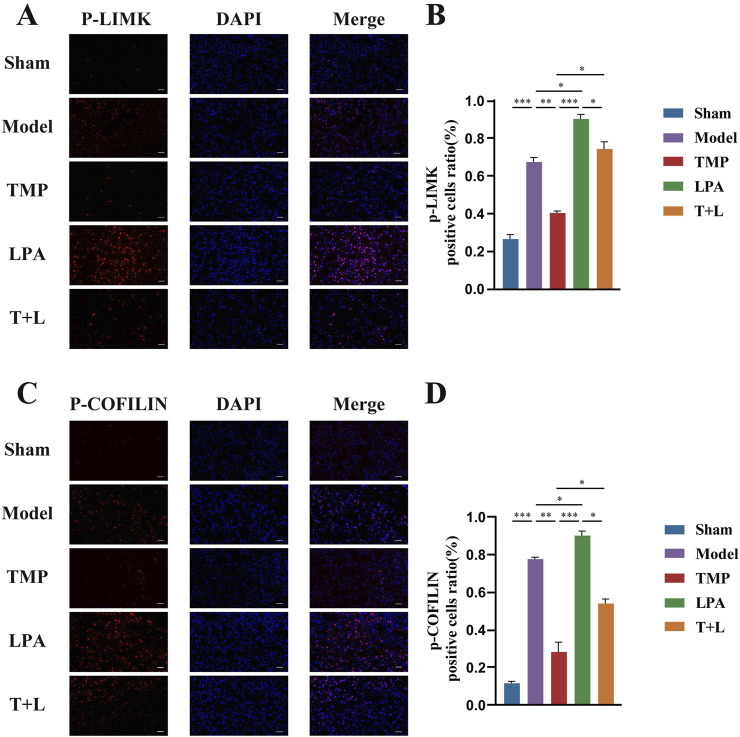
Immunofluorescence staining results of P-LIMK and P-COFILIN in different groups. **(A)** Immunofluorescence images for P-LIMK. **(B)** Quantification of P-LIMK positive cells. **(C)** Immunofluorescence images for P-COFILIN. **(D)** Quantification of P-COFILIN positive cells n = 5. Scale bar = 50 μm *P < 0.05, **P < 0.01, ***P < 0.001.

Figure 7A shows the representative bands of pathway proteins. The protein expression of the pathway proteins in model group was higher than that in sham group (Figures 7B–E; *P* < 0.001). The expression of pathway proteins was decreased after TMP intervention (Figures 7B–E; *P* < 0.05). The expression of pathway proteins was increased after the intervention of lysophosphatidic acids (LPA), an agonist of RhoA/ROCK signaling pathway (Figures 7B–E; *P* < 0.05). The levels of pathway proteins was significantly higher after the intervention of both TMP and LPA compared to TMP alone (Figures 7B–E; *P* < 0.05). The results of immunofluorescence were similar to Western blot results (Figures 8, 9; *P* < 0.05). TMP group had less positive cells than model group and TMP + LPA group had more positive cells than TMP group, suggesting that TMP may exert its neuroprotective effect through the RhoA/ROCK signaling pathway. The intervention of LPA could weaken the neuroprotective effect of TMP.

### 3.7 TMP upregulated neuroplasticity related proteins in rats after CIRI

**FIGURE 10:**
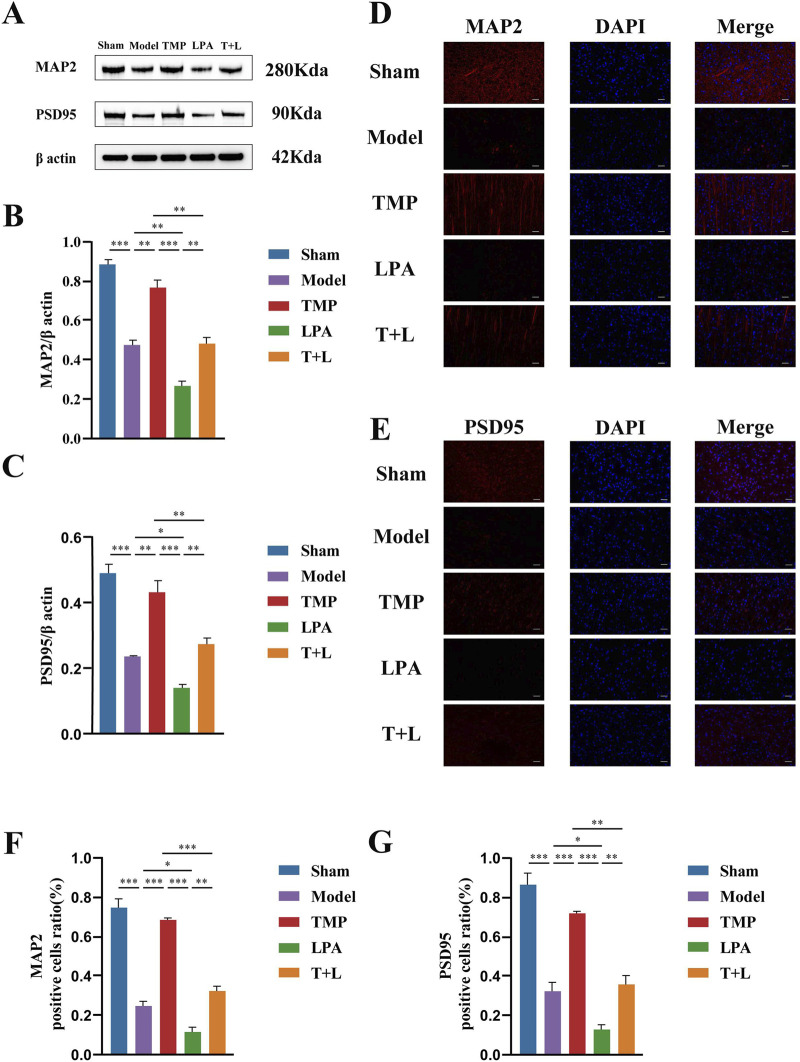
Western blot and immunofluorescence staining results of MAP2 and PSD95 in different groups. **(A)** Images of MAP2, PSD95 and β actin bands. **(B)** MAP2 protein expression analysis. **(C)** PSD95 protein expression analysis. **(D)** P-LIMK protein expression analysis.**(E)** P-COFILIN protein expression analysis. **(F)** Quantification of MAP2 positive cells. **(G)** Quantification of PSD95 positive cells *n* = 3. Scale bar = 50 µm *P < 0.05, **P < 0.01, ***P < 0.001.

To test the effect of TMP on neuroplasticity, we assessed synaptic and dendritic related proteins. Figure 10A shows the representative bands of proteins. Western blot results showed the expression of the synaptic plasticity-related protein PSD95 and dendritic plasticity-related protein MAP2 was significantly reduced in the model group compared to the sham group (Figures 10B,C; *P* < 0.001). The expression of PSD95 and MAP2 in the TMP group was significantly increased compared to the model group (Figures 10B,C; *P* < 0.01). However, the expression of PSD95 and MAP2 in the LPA group was reduced compared to the model group (Figures 10B,C; *P* < 0.05). Additionally, the expression of PSD95 and MAP2 were decreased in the TMP + LPA group compared to the TMP group (Figures 10B,C; *P* < 0.01). The trend observed in the immunofluorescence staining results was similar to that in the Western blot (Figures 10D–G; *P* < 0.05).

### 3.8 TMP promoted the regeneration of synapses and dendrites in rats after CIRI

**FIGURE 11:**
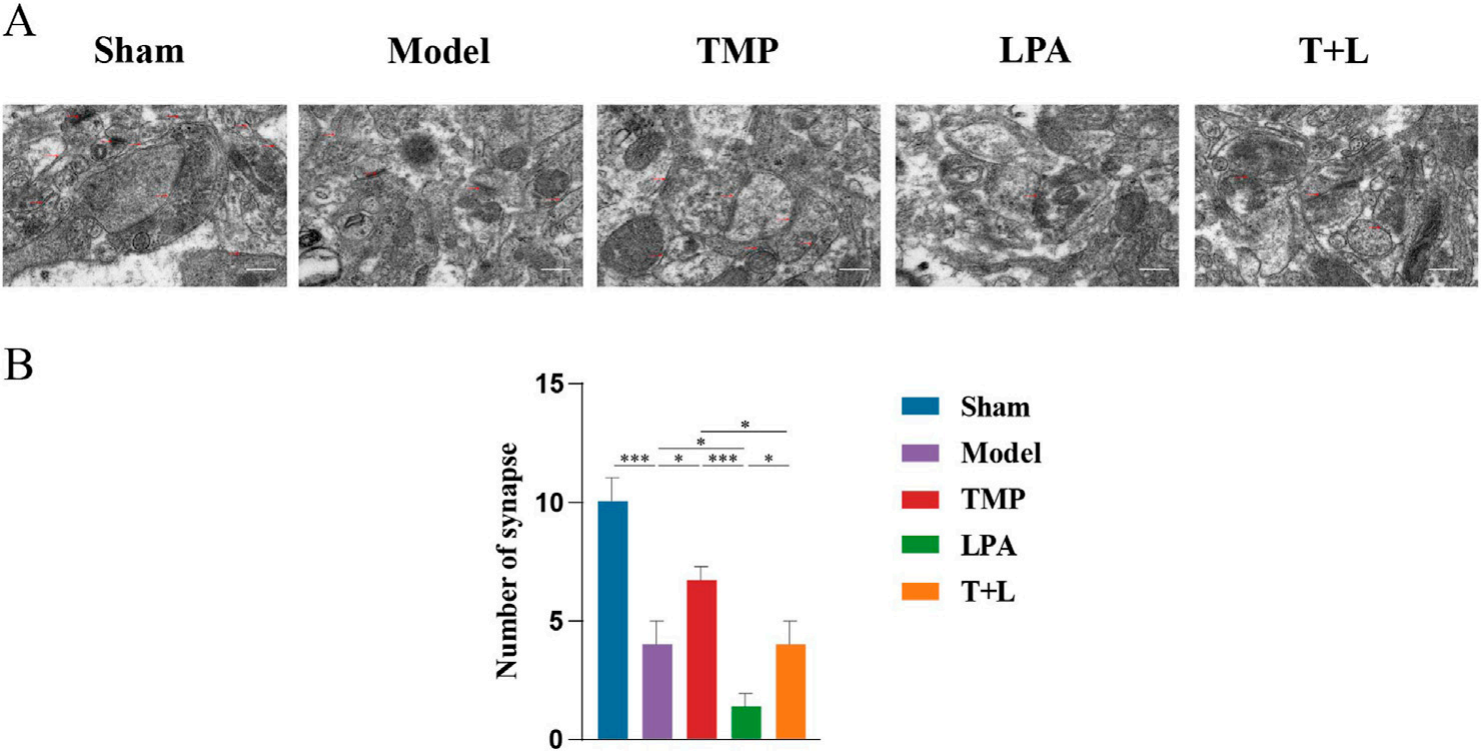
Morphological changes of synapses and dendrites. **(A)** Representative images for transmission electron microscopy of synapses. **(B)** Number of synapses *n* = 5. Scale bar = 50 µm *P < 0.05, ***P < 0.001.

To further verify the relationship between TMP and neuroplasticity, we observed the morphological changes of synapses and dendrites. Transmission electron microscopy (TEM) results showed that the sham group exhibited complete synaptic morphology. Model, TMP, LPA and TMP + LPA groups displayed significant synaptic disruption, fusion of synaptic gaps, and loss of vesicles (Figure 11A). Statistical analysis showed that the model group had fewer synapses than the sham group (Figure 11B; *P* < 0.001). The TMP group had more synapses than model group (Figure 11B; *P* < 0.05). In contrast, the LPA group had fewer synapses than the model group (Figure 11B; *P* < 0.05). Additionally, the number of synapses was significantly lower in the TMP + LPA group compared to the TMP group (Figure 11B; *P* < 0.05).

**FIGURE 12:**
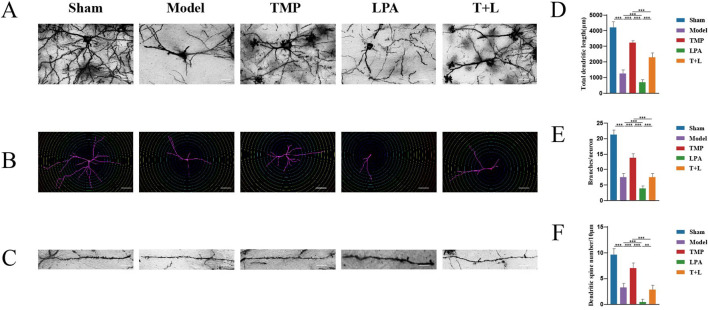
Morphological changes of synapses and dendrites. **(A)** Images for Golgi staining. **(B)** Images of dendrites via Sholl analysis. **(C)** Imagesof dendritic spines. **(D)** Total dendritic length in different groups. **(E)** Number of dendrites distributed on each neuron. **(F)** Number of dendritic spines *n* = 5. Scale bar = 50 µm *P < 0.05, **P < 0.01, ***P < 0.001.

Sholl analysis was used to measure the total dendritic length, number of dendrites per neuron, and number of dendritic spines per 10 µm of dendrites (Figures 12A–C). Statistical analysis showed that the model group had shorter dendrites, fewer branches, and fewer dendritic spines compared to the sham group (Figures 12D–F; *P* < 0.001). The TMP group had longer dendrites, more branches, and more dendritic spines than the model group (Figures 12D–F; *P* < 0.05). In contrast, the LPA group had shorter dendrites, less branches and dendritic spines compared to the model group (Figures 12D–F; *P* < 0.05). The TMP + LPA group had shorter dendrites, fewer branches, and fewer dendritic spines than the TMP group (Figures 12D–F; *P* < 0.001).

## 4 Discussion

The incidence of stroke continues to rise annually, impacting up to one in five individuals in some high-income countries and nearly half of the population in low-income countries (Hilkens et al., 2024). t-PA remains the only therapeutic agent for acute ischemic stroke. However, its efficacy is limited by a narrow therapeutic window, underscoring the urgent need for more effective treatments with fewer side effects (Fonarow et al., 2014). Therapeutic interventions during the subacute phase not only promotes functional recovery, but also reduces the risk of stroke recurrence. Implementing effective secondary prevention strategies in patients experiencing a first stroke or transient ischemic attack could potentially reduce the overall stroke burden by up to 25% (Hankey, 2014).

Natural products, especially active compounds derived from TCM, have demonstrated therapeutic effects in various diseases (Zhu et al., 2022). TMP, as a herbal extract, has been utilized for over two millennia in treating numerous ailments. TMP has shown efficacy in managing conditions such as stroke (Chen and Chen, 1992), coronary heart disease (Zhang et al., 2016), diabetes mellitus (Yang et al., 2011), cancer (Chen et al., 2013), and kidney injury (Gong et al., 2013). TMP can effectively cross BBB, making it a valuable agent in treating CNS disorders. Clinically, TMP has been employed in the management of CIRI for nearly 5 decades. In the past 2 decades, research on TMP’s effects on cerebral ischemia-reperfusion injury (CIRI) has gained significant attention. Ding et al. demonstrated that TMP could reduce oxygen and glucose deprivation (OGD)-induced neuronal death (Ding et al., 2019). Similarly, Gong et al. found that TMP enhances neural function recovery post-CIRI by restoring BBB integrity and function of the BBB (Gong et al., 2019). Our study corroborates these findings, showing that TMP promotes neurological function recovery as well as neuronal repair following CIRI in rats.

To elucidate the molecular mechanisms underlying TMP’s ameliorative effects on CIRI, we conducted mRNA sequencing on the peri-infarct tissues. DEGs analysis revealed significant upregulation of RhoA, ROCK2 and downstream genes such as LIMK1 and Cofilin1 in the model group compared to the sham group. TMP intervention markedly downregulated these genes. Heatmap and volcano plot analyses indicated distinct clustering patterns of RhoA/ROCK pathway-related genes between the model and TMP-treated groups. Gene Ontology (GO) enrichment analysis highlighted the significant association of RhoA and ROCK with neuroplasticity. Additionally, Gene Set Enrichment Analysis (GSEA) linked the RhoA/ROCK pathway to various biological processes. Specifically, RhoA influences cytoskeletal remodeling and postsynaptic membrane stability through ROCK activation, which subsequently phosphorylates and regulates downstream effectors like LIMK and Cofilin. Under pharmacological stimulation (e.g., cocaine and amphetamine), RhoA/ROCK signaling impacts synaptic connection formation, synaptic transmission efficiency, and neuronal excitability (Lepack et al., 2020; Ferreira et al., 2024). Moreover, it modulates calcium signaling’s regulatory effects on synaptic plasticity by adjusting the coupling efficiency of calcium ion transport proteins with the actin cytoskeleton (Zonouzi et al., 2011). KEGG pathway analysis further indicated a significant association between TMP intervention and the Ras signaling pathway. GSEA results also connected the RhoA/ROCK pathway with multiple other pathways. RhoA activation of ROCK1/2 inhibits cofilin’s depolymerization activity via LIMK phosphorylation, affecting actin stability. This regulation is crucial in cocaine addiction-related signaling and may influence synaptic transmission and dendritic spine morphology remodeling through CREB and dopamine receptor pathways (Tropea et al., 2022; Tropea et al., 2024). Additionally, sphingolipid and ether lipid metabolism play vital roles in cell membrane structure and signaling, interacting with RhoA/ROCK-mediated cytoskeletal remodeling (Incontro et al., 2025). The upregulation of steroid hormone and retinol metabolism may further activate the RhoA pathway, synergizing with neuronal synaptic adaptations (Lee et al., 2024; Carazo et al., 2021). Notably, the downregulation of the phagosome pathway suggests reduced immune stress following TMP intervention, aiding in maintaining neuronal function homeostasis and enhancing plasticity (Martinelli et al., 2021). Combined with the results of differential gene screening, GO analysis, and KEGG analysis, we proposed that RhoA and ROCK2 may be the pathogenic genes of CIRI, and TMP may promote nerve regeneration in CIRI rats through the RhoA/ROCK2 signaling pathway.

RhoA/ROCK pathway is implicated in various diseases, including ovarian cancer (Wei et al., 2021), chronic headache (Jing et al., 2019), traumatic brain injury (Mulherkar and Tolias, 2020), Parkinson’s disease (Iyer et al., 2021), stroke (Lu et al., 2023)and other neurological disorders. Numerous studies have demonstrated that inhibition of the RhoA/ROCK signaling pathway promotes neuroregeneration. For instance, Ding et al. demonstrated that hydrogen sulfide facilitates the phenotypic conversion of astrocytes to the A2 phenotype post-CIRI by inhibition of the RhoA/ROCK signaling pathway, thereby enhancing neurological recovery (Ding et al., 2024). Similarly, Yuan et al. reported that Xueshuantong injection promotes neuroregeneration by activation of the PI3K/AKT/mTOR signaling pathway and inhibiting RhoA/ROCK pathway, thereby enhancing synaptic plasticity after focal CIRI in rats (Yuan et al., 2022).

To further investigate TMP’s neuroprotective and neuroregenerative effects via the inhibition of the RhoA/ROCK pathway, we employed lysophosphatidic acid (LPA) as an agonist to activate this pathway (Sawada et al., 2002). Western blot and immunofluorescence staining analyses revealed that TMP downregulated the expression of RhoA, ROCK, P-LIMK, and P-Cofilin. Western blot and immunofluorescence staining analyses revealed that TMP downregulated the expression of RhoA, ROCK, phosphorylated LIMK (P-LIMK), and phosphorylated Cofilin (P-Cofilin) (Yang et al., 2017). Conversely, the addition of LPA attenuated TMP’s inhibitory effect on the RhoA/ROCK pathway, consistent with other studies (Li et al., 2022). Conversely, the addition of LPA attenuated TMP’s inhibitory effect on the RhoA/ROCK pathway, consistent with other studies (Levy et al., 2015), and MAP2, a dendritic plasticity-related protein sensitive to ischemia, were both upregulated by TMP treatment (Li et al., 1998). This upregulation was accompanied by enhanced synaptic and dendritic morphology regeneration. However, these effects were diminished upon LPA addition. The findings cumulatively indicate that TMP exerts its neuroprotective effects against CIRI primarily through modulation of the RhoA/ROCK signaling cascade. While our data support the involvement of the RhoA/ROCK2 pathway in TMP’s neuroprotection, whether TMP directly binds to RhoA/ROCK or their upstream regulators (e.g., GEFs like p115‐RhoGEF or LARG) remains unclear. Future studies employing biophysical assays (e.g., SPR) and genetic manipulation (e.g., GEF‐knockout models) are needed to identify the direct target. Additionally, the crosstalk between TMP and parallel pathways (e.g., MAPK/NF‐κB) warrants investigation to fully elucidate its mechanism.

## 5 Conclusion

Our findings indicate that TMP exerts neuroprotective and neuroregenerative effects in CIRI by inhibiting the RhoA/ROCK signaling pathway. By downregulating key components of this pathway, TMP promotes synaptic and dendritic plasticity, thereby facilitating neurological recovery. These insights not only enhance our understanding of TMP’s therapeutic mechanisms but also highlight the RhoA/ROCK pathway as a promising target for developing novel treatments for CIRI and related neurological disorders.

## Data Availability

The data presented in the study are deposited in the NCBI repository, accession number PRJNA1261237.

## Abbreviations

TMP, Tetramethylpyrazine; TCM, traditional Chinese medicine; BBB, blood-brain barrier; CNS, central nervous system; CIRI, cerebral ischemia-reperfusion injury; OGD, oxygen-glucose deprivation; MCAO/R, middle cerebral artery occlusion/reperfusion; MWM, Morris water maze; LPA, lysophosphatidic acid; GO, Gene Ontology; KEGG, Kyoto Encyclopedia of Genes and Genomes.

## References

[B1] Allen C. Srivastava K. Bayraktutan U. (2010). Small GTPase RhoA and its effector rho kinase mediate oxygen glucose deprivation-evoked *in vitro* cerebral barrier dysfunction. Stroke 41 (9), 2056–2063. 10.1161/STROKEAHA.109.574939 20651275

[B2] Bieber M. Gronewold J. Scharf A. C. Schuhmann M. K. Langhauser F. Hopp S. (2019). Validity and reliability of neurological scores in mice exposed to middle cerebral artery occlusion. Stroke 50 (10), 2875–2882. 10.1161/STROKEAHA.119.026652 31412755

[B3] Brown C. E. Murphy T. H. (2008). Livin' on the edge: imaging dendritic spine turnover in the peri-infarct zone during ischemic stroke and recovery. Neurosci. Rev. J. Bringing Neurobiol. Neurol. Psychiatry 14 (2), 139–146. 10.1177/1073858407309854 18039977

[B4] Campbell B. C. V. De Silva D. A. Macleod M. R. Coutts S. B. Schwamm L. H. Davis S. M. (2019). Ischaemic stroke. Nat. Rev. Dis. Prim. 5 (1), 70. 10.1038/s41572-019-0118-8 31601801

[B5] Carazo A. Macáková K. Matoušová K. Krčmová L. K. Protti M. Mladěnka P. (2021). Vitamin A update: forms, sources, Kinetics, detection, function, deficiency, therapeutic use and toxicity. Nutrients 13 (5), 1703. 10.3390/nu13051703 34069881 PMC8157347

[B6] Carlson A. P. Hänggi D. Macdonald R. L. Shuttleworth C. W. (2020). Nimodipine reappraised: an old drug with a future. Curr. Neuropharmacol. 18 (1), 65–82. 10.2174/1570159X17666190927113021 31560289 PMC7327937

[B7] Chen K. J. Chen K. (1992). Ischemic stroke treated with Ligusticum chuanxiong. Chin. Med. J. 105 (10), 870–873. 1291208

[B8] Chen Z. Pan X. Georgakilas A. G. Chen P. Hu H. Yang Y. (2013). Tetramethylpyrazine (TMP) protects cerebral neurocytes and inhibits glioma by down regulating chemokine receptor CXCR4 expression. Cancer Lett. 336 (2), 281–289. 10.1016/j.canlet.2013.03.015 23523616

[B9] Chen Z. Zhang C. Gao F. Fu Q. Fu C. He Y. (2018). A systematic review on the rhizome of Ligusticum chuanxiong Hort. (Chuanxiong). Food Chem. Toxicol. 119, 309–325. 10.1016/j.fct.2018.02.050 29486278

[B10] Ding Y. Du J. Cui F. Chen L. Li K. (2019). The protective effect of ligustrazine on rats with cerebral ischemia-reperfusion injury via activating PI3K/Akt pathway. Hum. and Exp. Toxicol. 38 (10), 1168–1177. 10.1177/0960327119851260 31250662

[B11] Ding Y. Fang F. Liu X. Sheng S. Li X. Yin X. (2024). H(2)S regulates the phenotypic transformation of astrocytes following cerebral ischemia/reperfusion via inhibiting the RhoA/ROCK pathway. Mol. Neurobiol. 61 (6), 3179–3197. 10.1007/s12035-023-03797-8 37978158

[B12] Fan F. Yang L. Li R. Zou X. Li N. Meng X. (2020). Salidroside as a potential neuroprotective agent for ischemic stroke: a review of sources, pharmacokinetics, mechanism and safety. Biomed. and Pharmacother. 129, 110458. 10.1016/j.biopha.2020.110458 32603893

[B13] Feng X. F. Li M. C. Lin Z. Y. Li M. Z. Lu Y. Zhuang Y. M. (2023). Tetramethylpyrazine promotes stroke recovery by inducing the restoration of neurovascular unit and transformation of A1/A2 reactive astrocytes. Front. Cell. Neurosci. 17, 1125412. 10.3389/fncel.2023.1125412 37051111 PMC10083399

[B14] Ferreira M. Carneiro P. Costa V. M. Carvalho F. Meisel A. Capela J. P. (2024). Amphetamine and methylphenidate potential on the recovery from stroke and traumatic brain injury: a review. Rev. Neurosci. 35 (7), 709–746. 10.1515/revneuro-2024-0016 38843463

[B15] Fonarow G. C. Zhao X. Smith E. E. Saver J. L. Reeves M. J. Bhatt D. L. (2014). Door-to-needle times for tissue plasminogen activator administration and clinical outcomes in acute ischemic stroke before and after a quality improvement initiative. Jama 311 (16), 1632–1640. 10.1001/jama.2014.3203 24756513

[B16] GBD 2019 Stroke Collaborators (2021). Global, regional, and national burden of stroke and its risk factors, 1990-2019: a systematic analysis for the Global Burden of Disease Study 2019. Lancet Neurol. 20 (10), 795–820. 10.1016/S1474-4422(21)00252-0 34487721 PMC8443449

[B17] Gong P. Zhang Z. Zou Y. Tian Q. Han S. Xu Z. (2019). Tetramethylpyrazine attenuates blood-brain barrier disruption in ischemia/reperfusion injury through the JAK/STAT signaling pathway. Eur. J. Pharmacol. 854, 289–297. 10.1016/j.ejphar.2019.04.028 31004602

[B18] Gong X. Wang Q. Tang X. Wang Y. Fu D. Lu H. (2013). Tetramethylpyrazine prevents contrast-induced nephropathy by inhibiting p38 MAPK and FoxO1 signaling pathways. Am. J. Nephrol. 37 (3), 199–207. 10.1159/000347033 23446291

[B19] Hacke W. Kaste M. Bluhmki E. Brozman M. Dávalos A. Guidetti D. (2008). Thrombolysis with alteplase 3 to 4.5 hours after acute ischemic stroke. N. Engl. J. Med. 359 (13), 1317–1329. 10.1056/NEJMoa0804656 18815396

[B20] Hankey G. J. (2014). Secondary stroke prevention. Lancet Neurol. 13 (2), 178–194. 10.1016/S1474-4422(13)70255-2 24361114

[B21] Hilkens N. A. Casolla B. Leung T. W. de Leeuw F. E. (2024). Stroke. Lancet 403 (10446), 2820–2836. 10.1016/S0140-6736(24)00642-1 38759664

[B22] Incontro S. Musella M. L. Sammari M. Di Scala C. Fantini J. Debanne D. (2025). Lipids shape brain function through ion channel and receptor modulations: physiological mechanisms and clinical perspectives. Physiol. Rev. 105 (1), 137–207. 10.1152/physrev.00004.2024 38990068

[B23] Iyer M. Subramaniam M. D. Venkatesan D. Cho S. G. Ryding M. Meyer M. (2021). Role of RhoA-ROCK signaling in Parkinson’s disease. Eur. J. Pharmacol. 894, 173815. 10.1016/j.ejphar.2020.173815 33345850

[B24] Jahani V. Kavousi A. Mehri S. Karimi G. (2018). Rho kinase, a potential target in the treatment of metabolic syndrome. Biomed Pharmacother. 106, 1024–1030. 10.1016/j.biopha.2018.07.060 30119167

[B25] Jing F. Zhang Y. Long T. He W. Qin G. Zhang D. (2019). P2Y12 receptor mediates microglial activation via RhoA/ROCK pathway in the trigeminal nucleus caudalis in a mouse model of chronic migraine. J. Neuroinflammation 16 (1), 217. 10.1186/s12974-019-1603-4 31722730 PMC6854723

[B26] Kim D. Langmead B. Salzberg S. L. (2015). HISAT: a fast spliced aligner with low memory requirements. Nat. Methods 12 (4), 357–360. 10.1038/nmeth.3317 25751142 PMC4655817

[B27] Lang P. Gesbert F. Delespine-Carmagnat M. Stancou R. Pouchelet M. Bertoglio J. (1996). Protein kinase a phosphorylation of RhoA mediates the morphological and functional effects of cyclic AMP in cytotoxic lymphocytes. EMBO J. 15 (3), 510–519. 10.1002/j.1460-2075.1996.tb00383.x 8599934 PMC449969

[B28] Langmead B. Salzberg S. L. (2012). Fast gapped-read alignment with Bowtie 2. Nat. methods 9 (4), 357–359. 10.1038/nmeth.1923 22388286 PMC3322381

[B29] Lee B. H. Eid R. S. Hodges T. E. Barth C. Galea L. A. M. (2024). Leveraging research into sex differences and steroid hormones to improve brain health. Nat. Rev. Endocrinol. 21, 214–229. 10.1038/s41574-024-01061-0 39587332

[B30] Lepack A. E. Werner C. T. Stewart A. F. Fulton S. L. Zhong P. Farrelly L. A. (2020). Dopaminylation of histone H3 in ventral tegmental area regulates cocaine seeking. Science 368 (6487), 197–201. 10.1126/science.aaw8806 32273471 PMC7228137

[B31] Levy J. M. Chen X. Reese T. S. Nicoll R. A. (2015). Synaptic consolidation normalizes AMPAR quantal size following MAGUK loss. Neuron 87 (3), 534–548. 10.1016/j.neuron.2015.07.015 26247861 PMC4596923

[B32] Li B. Dewey C. N. (2011). RSEM: accurate transcript quantification from RNA-Seq data with or without a reference genome. BMC Bioinforma. 12, 323. 10.1186/1471-2105-12-323 PMC3163565 21816040

[B33] Li M. Zhang L. Liu X. Wang G. Lu J. Guo J. (2022). Inhibition of Rho/ROCK signaling pathway participates in the cardiac protection of exercise training in spontaneously hypertensive rats. Sci. Rep. 12 (1), 17903. 10.1038/s41598-022-22191-3 36284153 PMC9596711

[B34] Li R. Li Y. Kristiansen K. Wang J. (2008). SOAP: short oligonucleotide alignment program. Bioinforma. Oxf. Engl. 24 (5), 713–714. 10.1093/bioinformatics/btn025 18227114

[B35] Li Y. Jiang N. Powers C. Chopp M. (1998). Neuronal damage and plasticity identified by microtubule-associated protein 2, growth-associated protein 43, and cyclin D1 immunoreactivity after focal cerebral ischemia in rats. Stroke 29 (9), 1972–1981. 10.1161/01.str.29.9.1972 9731626

[B36] Li Y. Tao T. Song D. He T. Liu X. (2021). Effects of Xuefu Zhuyu Granules on patients with stable coronary heart disease: a double-blind, randomized, and placebo-controlled study. Oxid. Med. Cell. Longev. 2021, 8877296. 10.1155/2021/8877296 34326921 PMC8302386

[B37] Lin J. Hao C. Gong Y. Zhang Y. Li Y. Feng Z. (2021). Effect of tetramethylpyrazine on neuroplasticity after transient focal cerebral ischemia reperfusion in rats. Evid Based Complement Alternat Med. 2021, 1587241. 10.1155/2021/1587241 33531914 PMC7834793

[B38] Longa E. Z. Weinstein P. R. Carlson S. Cummins R. (1989). Reversible middle cerebral artery occlusion without craniectomy in rats. Stroke 20 (1), 84–91. 10.1161/01.str.20.1.84 2643202

[B39] Love M. I. Huber W. Anders S. (2014). Moderated estimation of fold change and dispersion for RNA-seq data with DESeq2. Genome Biol. 15 (12), 550. 10.1186/s13059-014-0550-8 25516281 PMC4302049

[B40] Lu W. Chen Z. Wen J. (2023). The role of RhoA/ROCK pathway in the ischemic stroke-induced neuroinflammation. Biomed Pharmacother. 165, 115141. 10.1016/j.biopha.2023.115141 37437375

[B41] Margaill I. Parmentier S. Callebert J. Allix M. Boulu R. G. Plotkine M. (1996). Short therapeutic window for MK-801 in transient focal cerebral ischemia in normotensive rats. J. Cereb. Blood Flow. Metab. 16 (1), 107–113. 10.1097/00004647-199601000-00013 8530543

[B42] Martinelli S. Anderzhanova E. A. Bajaj T. Wiechmann S. Dethloff F. Weckmann K. (2021). Stress-primed secretory autophagy promotes extracellular BDNF maturation by enhancing MMP9 secretion. Nat. Commun. 12 (1), 4643. 10.1038/s41467-021-24810-5 34330919 PMC8324795

[B43] Matsui T. Amano M. Yamamoto T. Chihara K. Nakafuku M. Ito M. (1996). Rho-associated kinase, a novel serine/threonine kinase, as a putative target for small GTP binding protein Rho. EMBO J. 15 (9), 2208–2216. 10.1002/j.1460-2075.1996.tb00574.x 8641286 PMC450144

[B44] Mokin M. Ansari S. A. Mctaggart R. A. Bulsara K. R. Goyal M. Chen M. (2019). Indications for thrombectomy in acute ischemic stroke from emergent large vessel occlusion (ELVO): report of the SNIS Standards and Guidelines Committee. J. Neurointerventional Surg. 11 (3), 215–220. 10.1136/neurintsurg-2018-014640 30610069

[B45] Mulherkar S. Tolias K. F. (2020). RhoA-ROCK signaling as a therapeutic target in traumatic brain injury. Cells 9 (1), 245. 10.3390/cells9010245 31963704 PMC7016605

[B46] Nakagawa O. Fujisawa K. Ishizaki T. Saito Y. Nakao K. Narumiya S. (1996). ROCK-I and ROCK-II, two isoforms of Rho-associated coiled-coil forming protein serine/threonine kinase in mice. FEBS Lett. 392 (2), 189–193. 10.1016/0014-5793(96)00811-3 8772201

[B47] Ni X. Ni X. Liu S. Guo X. (2013). Medium- and long-term efficacy of ligustrazine plus conventional medication on ischemic stroke: a systematic review and meta-analysis. J. Tradit. Chin. Med. 33 (6), 715–720. 10.1016/s0254-6272(14)60002-9 24660601

[B48] Sawada K. Morishige K. Tahara M. Ikebuchi Y. Kawagishi R. Tasaka K. (2002). Lysophosphatidic acid induces focal adhesion assembly through Rho/Rho-associated kinase pathway in human ovarian cancer cells. Gynecol. Oncol. 87 (3), 252–259. 10.1006/gyno.2002.6831 12468322

[B49] Shah F. A. Kury L. A. Li T. Zeb A. Koh P. O. Liu F. (2019). Polydatin attenuates neuronal loss via reducing neuroinflammation and oxidative stress in rat MCAO models. Front. Pharmacol. 10, 663. 10.3389/fphar.2019.00663 31293416 PMC6606791

[B50] Shao H. He X. Zhang L. Du S. Yi X. Cui X. (2021). Efficacy of ligustrazine injection as adjunctive therapy in treating acute cerebral infarction: a systematic review and meta-analysis. Front. Pharmacol. 12, 761722. 10.3389/fphar.2021.761722 34880757 PMC8646035

[B51] Shao Z. Wang L. Liu S. Wang X. (2017). Tetramethylpyrazine protects neurons from oxygen-glucose deprivation-induced death. Med Sci Monit. 23, 5277–5282. 10.12659/msm.904554 29104282 PMC5685034

[B52] Takase H. Liang A. C. Miyamoto N. Hamanaka G. Ohtomo R. Maki T. (2018). Protective effects of a radical scavenger edaravone on oligodendrocyte precursor cells against oxidative stress. Neurosci. Lett. 668, 120–125. 10.1016/j.neulet.2018.01.018 29337010 PMC5829007

[B53] Tropea M. R. Gulisano W. Vacanti V. Arancio O. Puzzo D. Palmeri A. (2022). Nitric oxide/cGMP/CREB pathway and amyloid-beta crosstalk: from physiology to Alzheimer's disease. Free Radic. Biol. Med. 193 (Pt 2), 657–668. 10.1016/j.freeradbiomed.2022.11.022 36400326

[B54] Tropea M. R. Melone M. Li PUMA D. D. Vacanti V. Aceto G. Bandiera B. (2024). Blockade of dopamine D3 receptors improves hippocampal synaptic function and rescues age-related cognitive phenotype. Aging cell 23 (11), e14291. 10.1111/acel.14291 39236310 PMC11561665

[B55] Tsai T. H. Liang C. (2001). Pharmacokinetics of tetramethylpyrazine in rat blood and brain using microdialysis. Int. J. Pharm. 216 (1-2), 61–66. 10.1016/s0378-5173(01)00572-5 11274807

[B56] Virani S. S. Alonso A. Benjamin E. J. Bittencourt M. S. Callaway C. W. Carson A. P. (2020). Heart disease and stroke statistics-2020 update: a report from the American heart association. Circulation 141 (9), e139–e596. 10.1161/CIR.0000000000000757 31992061

[B57] Wang J. Wang L. Zhou H. Liang X. D. Zhang M. T. Tang Y. X. (2022a). The isolation, structural features and biological activities of polysaccharide from Ligusticum chuanxiong: a review. Carbohydr. Polym. 285, 118971. 10.1016/j.carbpol.2021.118971 35287839

[B58] Wang Q. Song L. J. Ding Z. B. Chai Z. Yu J. Z. Xiao B. G. (2022b). Advantages of Rho-associated kinases and their inhibitor fasudil for the treatment of neurodegenerative diseases. Neural Regen. Res. 17 (12), 2623–2631. 10.4103/1673-5374.335827 35662192 PMC9165373

[B59] Wang Y. Shi Y. Zhang X. Zou J. Liang Y. Tai J. (2019). A Chinese prescription chuanxiong Chatiao san for migraine: a systematic review and meta-analysis of randomized controlled trials. Evid Based Complement Alternat Med 2019, 2301680. 10.1155/2019/2301680 31467571 PMC6699287

[B60] Wei X. Lou H. Zhou D. Jia Y. Li H. Huang Q. (2021). TAGLN mediated stiffness-regulated ovarian cancer progression via RhoA/ROCK pathway. J. Exp. Clin. Cancer Res. 40 (1), 292. 10.1186/s13046-021-02091-6 34538264 PMC8451140

[B61] Xu H. Qin W. Hu X. Mu S. Zhu J. Lu W. (2018). Lentivirus-mediated overexpression of OTULIN ameliorates microglia activation and neuroinflammation by depressing the activation of the NF-κB signaling pathway in cerebral ischemia/reperfusion rats. J. Neuroinflammation 15 (1), 83. 10.1186/s12974-018-1117-5 29544517 PMC5856386

[B62] Yang G. Qian C. Wang N. Lin C. Wang Y. Wang G. (2017). Tetramethylpyrazine protects against oxygen-glucose deprivation-induced brain microvascular endothelial cells injury via rho/rho-kinase signaling pathway. Cell. Mol. Neurobiol. 37 (4), 619–633. 10.1007/s10571-016-0398-4 27380043 PMC11482156

[B63] Yang J. Li J. Lu J. Zhang Y. Zhu Z. Wan H. (2012). Synergistic protective effect of astragaloside IV-tetramethylpyrazine against cerebral ischemic-reperfusion injury induced by transient focal ischemia. J. Ethnopharmacol. 140 (1), 64–72. 10.1016/j.jep.2011.12.023 22207211

[B64] Yang Q. H. Liang Y. Xu Q. Zhang Y. Xiao L. Si L. Y. (2011). Protective effect of tetramethylpyrazine isolated from Ligusticum chuanxiong on nephropathy in rats with streptozotocin-induced diabetes. Phytomedicine. 18 (13), 1148–1152. 10.1016/j.phymed.2011.05.003 21665452

[B65] Yu B. Zhong F. M. P. Yao Y. Deng S. Q. Xu H. Q. Lu J. F. (2019). Synergistic protection of tetramethylpyrazine phosphate and borneol on brain microvascular endothelium cells injured by hypoxia. Am. J. Transl. Res. 11 (4), 2168–2180. 31105826 PMC6511760

[B66] Yuan Q. Wang F. J. Jia Z. Z. Zhang T. Sun J. Du X. Y. (2022). Xueshuantong injection combined with Salvianolate lyophilized injection improves the synaptic plasticity against focal cerebral ischemia/reperfusion injury in rats through PI3K/AKT/mTOR and RhoA/ROCK pathways. Brain Res. 1787, 147923. 10.1016/j.brainres.2022.147923 35461832

[B67] Zhang Y. Ma X. J. Guo C. Y. Wang M. m. Kou N. Qu H. (2016). Pretreatment with a combination of ligustrazine and berberine improves cardiac function in rats with coronary microembolization. Acta Pharmacol. Sin. 37 (4), 463–472. 10.1038/aps.2015.147 26924290 PMC4820796

[B68] Zhang Y. Q. Wu J. B. Yin W. Huang Z. J. (2020). Design, synthesis, and biological evaluation of ligustrazine/resveratrol hybrids as potential anti-ischemic stroke agents. Chin. J. Nat. Med. 18 (8), 633–640. 10.1016/S1875-5364(20)30076-5 32768171

[B69] Zhou Z. Lu J. Liu W. W. Manaenko A. Hou X. Mei Q. (2018). Advances in stroke pharmacology. Pharmacol. Ther. 191, 23–42. 10.1016/j.pharmthera.2018.05.012 29807056

[B70] Zhu Y. Ouyang Z. Du H. Wang M. Wang J. Sun H. (2022). New opportunities and challenges of natural products research: when target identification meets single-cell multiomics. Acta Pharm. Sin. B 12 (11), 4011–4039. 10.1016/j.apsb.2022.08.022 36386472 PMC9643300

[B71] Zonouzi M. Renzi M. Farrant M. Cull-Candy S. G. (2011). Bidirectional plasticity of calcium-permeable AMPA receptors in oligodendrocyte lineage cells. Nat. Neurosci. 14 (11), 1430–1438. 10.1038/nn.2942 21983683 PMC3204222

